# Ethics in mental health for early career psychiatrists

**DOI:** 10.1192/j.eurpsy.2023.202

**Published:** 2023-07-19

**Authors:** O. Kilic

**Affiliations:** Department of Psychiatry, Bezmialem Vakif University Faculty of Medicine, Istanbul, Türkiye

## Abstract

**Abstract:**

Professional attitudes and ethics skills of physicians are influenced in part by the lessons of medical training. Still, few medical schools and postgraduate training programs introduce a formal curriculum. There is also the hidden curriculum which is influenced by instructions that are implicitly learned by observation of others, the cultural climate, and the social norms. The hidden ethics curriculum in psychiatry resident programs was investigated with qualitative interviews. Patient autonomy (consent for admission, coercive treatments) and ethical problems that arise during the management of difficult patient populations (medical ills, substance users, and frequent fliers) were the most common themes. Psychiatric residents perceive a need for more education on ethical issues. The speaker will present the available knowledge on psychiatry residents’ perspectives and the current programs addressing ethics training. This talk is hoped to elicit discussion in preparation for future action and inform a roadmap for addressing ethics training and subsequent educational events during psychiatry undergraduate and postgraduate education.

**Disclosure of Interest:**

None Declared

